# Fitness Profiles of Elite Portuguese Rugby Union Players

**DOI:** 10.2478/hukin-2014-0051

**Published:** 2014-07-08

**Authors:** Luís Vaz, Tomaz Morais, Henrique Rocha, Nic James

**Affiliations:** 1 Research Center in Sports Sciences, Health and Human Development (CIDESD), Portugal.; 2 Ex National Team Coach in Portuguese Rugby Federation, Portugal.; 3 London Sport Institute at Middlesex University, England.

**Keywords:** Rugby, physical and fitness tests, performance

## Abstract

The aim of this study was to describe the fitness profiles of senior elite Portuguese rugby players. Forty-six senior Portuguese rugby players, classified as backs (n=22; age 26.2±2.8) and forwards (n=24; age 26.7±2.9) were assessed during physical testing sessions carried out for the Portuguese National rugby team. The body composition, maximum strength and anaerobic capacity of players are hypothesized to be important physical characteristics as successful performance in rugby is predicated on the ability to undertake skilled behaviours both quickly and whilst withstanding large forces when in contact situations. No absolute differences were found between the backs and forwards for the speed performance variables although positional differences were found across all speeds when assessed relative to body mass since the forwards were significantly heavier. Coaches and the management team can use this information for monitoring progressive improvements in the physiological capacities of rugby players. These physical characteristics of elite rugby players provide normative profiles for specific positions and should form the basis of developmental programmes for adolescents.

## Introduction

Rugby is a high-intensity contact team sport that requires players to possess a wide range of physical attributes (Gabbett et al., 2009). There are two main types of a playing position in rugby, referred to as forwards and backs, with each likely to require different fitness levels and anthropometry due to different game demands. For example, the main responsibilities of the forward players are to gain and retain possession of the ball, usually in contact situations involving multiple players acting in unison. Hence, players in these positions are usually the biggest and strongest and take part in the set piece restarts scrum and line-out (James et al., 2005). Backs can vary a lot in terms of body height but tend to have low body fat and are faster and more agile than the forwards. Their role involves running quickly over greater distances where they try to create and convert point-scoring opportunities. Both types of players have to stop the opposition from running with the ball by tackling them, yet kicking is usually left to the backs.

A wide variety of performance tests have been used to measure the fitness characteristics of rugby players (Johnston and Gabbett, 2011; Lockie et al., 2012) but this breadth makes it difficult to compare between studies. However, Gabbett et al. (2007) reported that forwards were heavier and had larger skinfold measurements compared to backs and had slower times in change of direction tests as well as in the 20 and 40 m sprint performance. While forwards were heavier and slower than backs these characteristics were deemed suitable as they are required to be more combative than the backs.

The physical demands of competition have also been investigated through the use of time motion analysis (Cupples and O’Connor, 2011; Vaz et al., 2010), global positioning systems (McLellan et al., 2011; Suarez-Arrones et al., 2012) and the measurement of various physiological variables (Austin et al., 2011; Johnston et al., 2012). The development of notational analysis and the identification of key performance indicators have also provided further information into specific playing profiles of successful teams and individuals (Hughes and Bartlett, 2002).

It is commonly believed that physical preparation should reflect the degree to which each component of fitness is relied upon in competition. Nonetheless, few studies have examined the direct relationship between fitness testing measures and key performance indicators in competition. Thus comprehensive studies of physical characteristics of players, fitness requirements and movement patterns have contributed to the development of more effective conditioning programs (Alemdaroglu, 2012; Biscombe and Drewett, 2010). However, insufficient research has dealt with detailed assessment of physical demands of rugby in relation to the fitness tests used to measure these attributes.

Literature in physiological and anthropometric demands of rugby (Docherty et al., 1988; Elloumi et al., 2012; Higham et al., 2012) has been extensively researched, as numerous studies have been conducted on this subject, specifically using anthropometric data.

Some studies (Gabbett et al., 2007; Lopez-Segovia et al., 2011) have found significant correlations between fitness test results (e.g. vertical jump) and the attributes of agility skill execution of the player (r = 0.44) and between speed (e.g. sprint time: 10, 20 and 40 m) and offensive skills. These results are to some extent expected, as players with higher levels of fitness are likely to have an advantage in the performance of skills in competition. Further research, however, is required to quantify the direct relationships between physical fitness profiles and competition performance, providing coaches with specific attributes that contribute to the desired performance of key performance indicators on the field of play. To prevent overtraining and to ensure that the athletic training program will result in performance improvements, or at least the maintenance of performance standards, regular testing is suggested to be included as a vital component in the training program.

The research data from this study will help provide coaches with the necessary information to construct up to date training programs to stimulate or overload physiological rugby game conditions, without overtraining players. The aim of this study was to describe the fitness profiles of seniors’ elite Portuguese rugby players.

## Material and Methods

### Participants

Forty-six senior Portuguese rugby players classified as backs (n=22; age 26.2±2.8) and forwards (n=24; age 26.7±2.9) were assessed during physical testing of the Portuguese National rugby team. The players belonged to Portuguese and European clubs and took part in National and International competitions during the 2009/2010 season. They trained with their clubs on average 5 to 6 times per week i.e. 10 to 12 hours weekly. In the preparation for International competition they trained with the National team twice a day from Monday to Friday with a corresponding training volume of 4 to 6 hours per day.

The dietary intake was assessed and administrated by the Federation nutritionist who supervised all nutritional menus for the players. According to the medical staff none of the participants was taking medications or exhibited metabolic and/or endocrine dysfunctions that could impede or limit their ability to fully participate in the study. The study was conducted between October 2009 and March 2010 during the initial preparation period for the Division 1A 2009–2010 European Nations Cup (Georgia, Portugal, Spain, Russia. Romania and Germany), which also acted as the 2011 Rugby World Cup qualifying competition.

The participants, coaches and management of the National team approved the fitness protocol testing procedures and were notified that they could withdraw from the study at any time.

All participants gave informed consent and authority for the data to be used for research purposes.

The testing procedures were fully explained beforehand and on the day of testing. Measurements were taken by medical and qualified personnel fully trained to use the equipment. The study protocol was conformed to the declaration of Helsinki and was approved by the University ethics committee of the Research Center in Sport, Health and Human Development (Portugal).

### Study Design

All participants took part in a four day training and testing protocol as players of the Portuguese national rugby team with full access to the planned sessions and recuperation protocol undertaken with the usual pre-training diet. Participants were randomly divided into three groups consisting of approximately an equal number of players. At the outset all anthropometric measurements were taken for each participant three times, at the same time of the day, at the National Center of Medicine and Science in Sports, Lisbon (average temperature: October 19.0 ± 2 °C to March 15.1 ± 2º C).

For the field tests, participants underwent a standardised warm up (progressing from low to higher intensity activities) and a stretching routine. Players were encouraged to perform low intensity activities and stretches between trials to minimise reductions in performance. Upon completion of the respective tests, each group rotated until all tests were performed. The field testing session was concluded with participants performing the speed and multi-stage fitness test (VO_2_ max). To standardise conditions between the three groups, testing sessions were conducted on the same field at the same time of a day.

The same staff (Portuguese Federation) and kinanthropometry laboratory (FMH) were used for all tests for each group. Each participant was instructed and verbally encouraged to give a maximal effort during all tests.

### Fitness protocol

The validity of the tests selected for this study was confirmed earlier (Ross and Marfell-Jones, 1991). They were thought to be effective in that they satisfied the following criteria: i) specific and relevant to the needs of the sport; ii) repeatable and reliable - i.e. the same test would produce the same result in 2 athletes of the same fitness level; iii) easy and time efficient to conduct; iv) the results provided are easy to interpret and v) they can also substitute for a training session in the fitness aspect it measures.

### Day 1:

Anthropometric evaluation: body mass (kg); body height (cm); body fat percentage, sum of seven skinfolds (sum of triceps, biceps, subscapular, supra iliac, calf, thigh and abdominal skinfolds); girths (flexed upper arm, calf, subgluteal, mid high, knee and, fore-arm); breadths (humerus, femur), muscle mass and somatotype were measured with calibrated devices and all measurements were performed by the medical staff of a FMH kinanthropometry laboratory (Lohman et al., 1988; Ross and Marfell-Jones, 1991).

Maximal strength was assessed with a test of 3–5 RM - Testing (Argus et al., 2012; Crewther et al., 2009). This test measures the player’s ability to lift a sub-maximal load during 3 to 5 repetitions which allows to determine the subjects’ 1RM. If the participant is not able to complete 3 repetitions or if he performs more than 5 repetitions, the test has to be repeated after a 5 minute rest period.

In the front squat (Baker, 2009; Harrison and Bourke, 2009), the player has to fully bent the knees and hips until thighs are parallel to the floor. After that, knees and hips are extended until legs are straight. Weight belts were not allowed. In all of the other tests, the load and the number of repetitions performed were used to estimate the player′s 1 RM using the following formula:

$$\begin{array}{c} \mathrm{1RM}=\mathrm{Weight lifted}/(([\mathrm{Exp}(-0\mathrm{.055}\times \mathrm{Reps}\\ \mathrm{completed}))]\times \mathrm{41}\mathrm{.9}+\mathrm{52}\mathrm{.2}/\mathrm{100} \end{array}$$

### Day 2:

The speed (Green et al., 2011) and multistage fitness tests (Ramsbottom et al., 1988) were performed in day 2 of the protocol. Players had a minimum of a 20 minute warm up including a number of short maximal efforts prior to testing. Running speed of players was evaluated with a 10, 20, 30 40 and 50 m sprint effort using dual beam electronic timing gates (swift performance equipment). The timing gates were positioned 10, 20, 30, 40 and 50 m cross wind from a predetermined starting point. Players were instructed to run as fast as possible along the 50 m distance from a standing start.

On a synthetic track, participants commenced the test in their own time, with timing starting once the beams of the first (0 m) timing gate were broken.

The intra class correlation coefficient for test-retest reliability and typical error of measurement for this sprint test were 0.95 to 0.97, 1.8% to 1.2%, respectively.

Maximal aerobic power was estimated using the multi-stage fitness test (Ramsbottom et al., 1988). Players were required to run back and forth (i.e. shuttle run) along a 20 m track, keeping in time with a series of signals on a compact disk. The frequency of the audible signals (and hence, running speed) was progressively increased, until subjects reached volitional exhaustion. Maximal aerobic power (VO_2max_) was estimated using regression equations described by Ramsbottom et al. (1988).

### Statistical analysis

Data are expressed as means ± SD. Independent sample t-tests were performed to study differences between backs and forwards across all variables. Corresponding effect sizes (ES) were calculated and interpreted based on the following criteria: <0.20 = trivial; 0.20 to 0.59 = small; 0.60 to 1.19 = moderate, 1.20 to 2.0 = large, > 2.0 = very large (Hopkins, 2002).

In addition, all performance variables were normalised based on individual weight and re-analysed using the same statistical test. All data sets were tested for the assumptions corresponding to each statistical test and were analyzed using the statistical software SPSS for Windows, version 19.0 (SPSS Inc., Chicago, IL). The level of statistical significance was set at p<0.05.

## Results

The body mass (kg) of backs was significantly lower (t = −3.2, p < .001, ES = −1.04) than the one of forwards (Table 1) and they had played more International matches (t = 2.1, p < .05, ES = 0.80).

**Figure 1:**
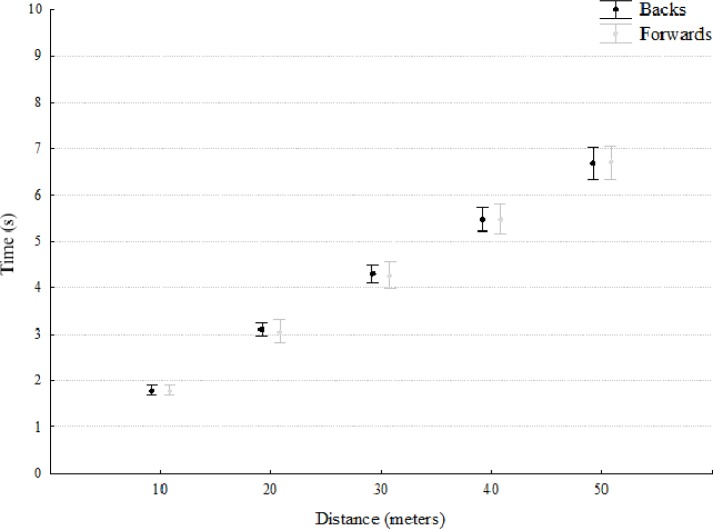
Accumulated speed performance

**Figure 2:**
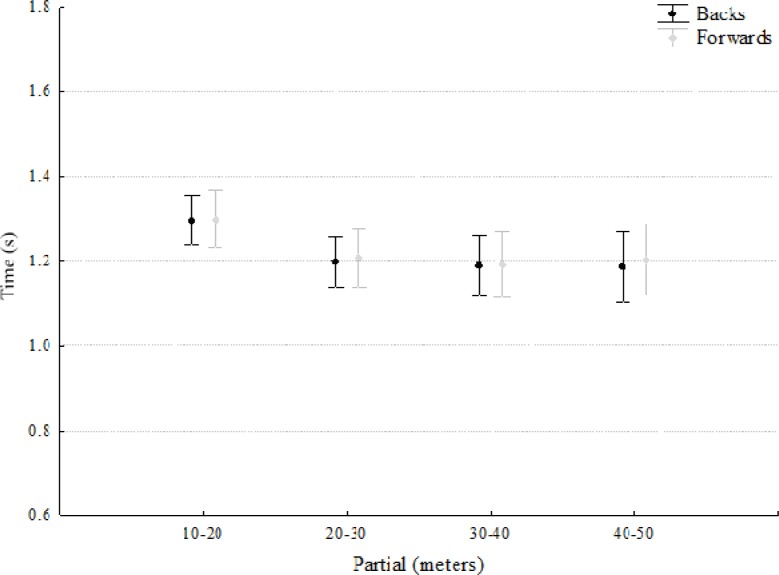
Partial speed performance

**Figure 3:**
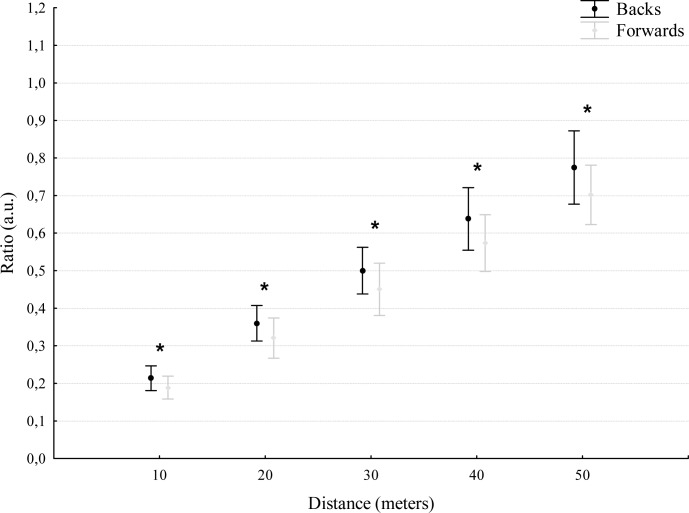
Speed performance in ratio variable/body mass *Statistic differences identified in ratio variable/body mass.

No differences were found between the backs and forwards for the speed performance variables (Figure 1 and 2). However, when these speed variables were adjusted for players’ weight, differences were found across all indicators between positions (10 m: t = 4.2, p<. 001, ES = 0.80; 20 m: t = 3.2, p < .01, ES = 0.79; 30 m: t = 3.1, p < .01, ES = 0.75; 40 m: t = 4.3, p< .01, ES = 0.81; and 50 m: t = 3.3, p< .01, ES = 0.82) (Figure 3).

The forwards had significantly higher values for 1RM bench press (t = −2.6, p < .05, ES = −0.55), 1RM Squat (t = −2.6, p <.05, ES = −1.03) and 1RM leg press (t = −2.6, p < .05, ES = −1.33; Table 3).

## Discussion

As previously found the forwards exhibited higher body mass than the backs; thought to be useful for their primary tasks which are to wrestle, physically compete and perform vertical jump actions to catch the ball (Quarrie et al., 2012). As a consequence, coaches commonly consider the forwards’ body mass as a key criterion to success in the players’ performance.

The backs had played in more international (15-a-side rugby union and seven’s) matches (30.7±18.9) than the forwards (17.0±15.1). A lower incidence of injuries or because many rugby players concurrently compete in 15-a-side rugby union as backs can help to understand this result. However, further research is required to establish the truth in these hypotheses as the relationship between experience and age does not seem to be well researched, particularly in terms of forwards and backs playing in international competitions.

Interestingly no absolute differences were found between backs and forwards in the speed test. Of course the ability to move quickly over various distances, starting from a variety of positions and speeds, is a key component in all players’ performances (Duthie et al., 2003), however, previous studies have found differences between backs and forwards although these appear to be more pronounced at greater distances, e.g. no differences were found for 10 m sprint times but significant differences for 40 m sprint times (T. J. Gabbett, 2012). This might be due to the different roles in competition, as backs have been shown to sprint longer and more frequently than forwards.

The maximum running speed of rugby players is usually measured over sprint distances of 30 – 50 m on the basis that players develop close to maximum running speeds over similar sprint distances during a game (Duthie, Pyne, Marsh et al., 2006). In the current study, a 50 m sprint test was employed to measure the player′s ability to develop acceleration and high running velocities. Examination of the 10–50 m sprint times revealed no significant differences between the forwards and backs.

Rugby forwards typically perform 10–15 short distance (10–20 m) sprints during a game, therefore, the initial acceleration over the first 10 m of a sprint may be a critical factor in their performance (Duthie, Pyne, Marsh et al., 2006). Thus, for rugby forwards, the ability to attain maximum speed quickly following a break from the opposition is an important performance requirement for this group.

Maximum running velocity in rugby players is usually achieved in the latter part of longer sprints of 30–50 m and there is a lack of research on the ability of rugby players to develop maximum running speed over these distances. Consequently, in the current study sprinting times were obtained over the 10–50 m sprint distances to reflect the development of maximum running speed in typical sprint distances during a match. However, it has previously been shown that body mass and body height of athletes influence sprint running performance (Gabbett, 2012).

In the current study when individual body mass was considered as a covariate, differences were found across all indicators between forwards and backs suggesting a significant influence of body mass on sprinting performance. Because of the typical body mass discrepancy between backs and forwards when viewed as groups, differences between these playing positions are always likely and will be primarily a result of different positional roles in competition. For example, backs have been shown to sprint longer and more frequently than forwards. At higher levels of competition, there may be more specific selection criteria for the performance requirements of positional groups. Acceleration and maximum running velocity sprint times measured over distances of 0–10, 20, 30, 40 and 50 m, appear to differentiate between forwards and backs. These differences may reflect the specific performance requirements of these positions and differences in anthropometric characteristics such as body mass. The ability to accelerate is an important quality for all players, but for backs, it represents specific characteristics or adaptation associated with the need to perform an increased number of shorter sprints during a match compared to the forwards.

Forwards are involved in more rucks, mauls, lineouts and scrums, which require greater body mass, body height, strength and power in order to be successful. In contrast, the backs’ primary role in beating the opposition in open play requires a combination of speed, acceleration and agility. For forwards, acceleration may be less important, given their higher involvement in the physical contact aspects of the game. Screening programs for the selection and monitoring of performance in rugby (forwards and backs) should include the evaluation of sprinting performance over the shorter distances (10 – 15 m) as the majority of sprint runs in forwards’ play involve the acceleration phase only.

Speed characteristics of the players in the present study are similar to those previously reported in the rugby union. Indeed, backs have been shown to be faster over distances greater than 30 m than forwards. A decreased ability to perform repeated sprints may reduce the involvement of the player in multiple rucks and open play, thus decreasing the number of activities completed.

In contrast, greater repeated sprint ability may increase the player’s involvement in more rucks, increasing the chance to receive the ball and the subsequent involvement in more tasks. Perhaps accounting for the backs performing better on the multi-stage fitness test in this study, forwards usually present higher body mass indexes and spend 12 to 13% of the total match time performing high-intensity work (Comyns et al., 2010) in order to compete for ball possession.

These specific playing position demands require the development of maximum muscle strength (Adendorff et al., 2004). Forwards are generally stronger than backs taking into account both upper and lower body due to fitness requirements in scrums and the higher frequency in which the forwards are involved in tackles and ruck situations (Quarrie et al., 2012). Additionally, as the playing level increases, strength also increases. Allometric scaling has been used to allow a more effective comparison of strength between forwards and backs (Crewther et al., 2009). However, only two studies have reported scaled strength values in contact team sport players, illustrating no differences between forwards and backs (Atkins, 2004). Further work is therefore required to compliment these findings and to establish trends and differences in relative strength within higher level players. As a consequence, rugby coaches should be focused on the players’ maximal force capacity, rate of force development, muscle coordination and stretch-shortening cycle development. Thus, the use of specific external loads should be considered to induce considerable neuromuscular and structural adaptations.

### Backs characteristics

- The backs role in beating the opposition in open play requires speed, acceleration and agility.
- Backs need to be able to beat the opposition in open play, thus are required to be fast and agile.
- Backline players need explosive leg power to be able to accelerate to create opportunities for the wings.
- Backs cover a greater distance than forwards during a game.

### Forward characteristics

- Forwards are generally taller, heavier and have higher body fat content than the backs with differences of ∼5%, ∼15% and ∼25%, respectively (Duthie, Pyne, Hopkins et al., 2006) .
- Typically, forwards have an endomorphic-mesomorphic physique compared to the backs (Olds, 2001).
- Forwards tend to have higher endomorphy and lower ectomorphy than backs, which is probably due to the strength demands placed upon them at the contact situation.
- Forwards are generally stronger than backs in both upper and lower body due to requirements of strength in scrums and the higher frequency in which the forwards are involved in tackles and ruck situations (Quarrie et al., 2012).

## Practical implications

Fitness testing is an effective way for evaluation of a current fitness level where the results of tests can also give a starting point for determining the intensity and volume of work required to ensure that progression occurs in a training cycle. Regular performance tests and data such as these will inform coaches, so that they have better understanding of what should be expected and what could be achieved with a rugby player over a season. The information should help coaches develop strategies, such as more specific periodisation and recovery, and to improve the decrements in performance during specific times of the year.

The relationships between physical characteristics and game behaviours highlight the importance of these characteristics in the performance of specific aspects of competition.

The physical characteristics of elite rugby union players provide normative profiles of specific positions, playing levels and should form the basis of development programmes for adolescents.

To prevent overtraining and to ensure that the athletic training program will result in performance improvements, or at least the maintenance of performance standards, it is suggested that regular performance tests are included as a component of the training program.

## Conclusions

Body mass and body composition as well as speed and repeated sprint ability appear to be important physical and fitness characteristics for superior performance in rugby. Regular testing of the motor potential is a must in team sport games as the result allow to direct the training process.

Body mass and sprint performance significantly differentiate backs and forwards in rugby.

Given the greater detail surrounding the effect physical characteristics have upon game performance, a player’s physical preparation can be specifically adjusted to improve attributes that will enhance the performance of their role within competition.

Future research should therefore employ programmes that encompass concurrent strength and conditioning and skill based training; which will provide insight into the long-term developmental changes in the attributes associated with elite performance.

## Figures and Tables

**Table 1:** Description of the players

	Backs *(n=22)*	Forwards *(n=24)*	t	p	ES
Body mass (kg)	88.0±11.4	100.7±12.9	−3.2	.003	−1.04
Body height (cm)	180.6±7.1	184.4±6.3	−1.6	.123	-
Age (years)	26.2±2.8	26.7±2.9	−0.5	.618	-
International matches	30.7±18.9	17.0±15.1	2.1	.042	0.80

**Table 2:** Maximum strength

	Backs	Forwards	t	p	ES
1RM Bench press	98.33±19.84	109.45±20.56	−2.6	.012	−0.55
1RM Squat	202.36±25.24	233.33±34.15	−2.6	.015	−1.03
1RM Leg press	505.91±63.10	583.33±85.38	−2.6	.015	−1.03
